# LINC00202 promotes retinoblastoma progression by regulating cell proliferation, apoptosis, and aerobic glycolysis through miR-204-5p/HMGCR axis

**DOI:** 10.1515/biol-2020-0047

**Published:** 2020-06-30

**Authors:** Aimin Wu, Xuewei Zhou, Linglong Mi, Jiang Shen

**Affiliations:** Department of Ophthalmology, Fenghua District People’s Hospital of Ningbo City, Ningbo, No. 36 Gongyuan Road, Fenghua District, Ningbo City, Zhejiang Province, 315500, China; Department of Ophthalmology, Ningbo Eye Hospital, Ningbo, China

**Keywords:** LINC00202, miR-204-5p, HMGCR, aerobic glycolysis, retinoblastoma

## Abstract

LINC00202 is a newly identified long noncoding RNA (lncRNA) and has been demonstrated to involve in the progression of retinoblastoma (RB). Here, we further explored the role and the underlying molecular mechanism of LINC00202 on RB malignant properties and glycolysis. LINC00202, microRNA (miR)-204-5p, and 3-hydroxy-3-methyl-glutaryl-coenzyme A reductase (HMGCR) mRNA were detected by a quantitative real-time polymerase chain reaction. Cell proliferation and apoptosis were analyzed using cell counting kit-8 assay and colony formation assay and flow cytometry, respectively. Glucose metabolism was calculated by measuring the extracellular acidification rate (ECRA). Western blot was used to detect the levels of HMGCR, ki67, pro-caspase-3, cleaved-caspase-3, and lactate dehydrogenase A chain (LDHA). The interaction between miR-204-5p and LINC00202 or HMGCR was analyzed by the dual-luciferase reporter assay. Murine xenograft model was established to conduct *in vivo* experiments. LINC00202 expression was upregulated in RB tumor tissues and LINC00202 knockdown inhibited RB cell proliferation, glycolysis, and stimulated apoptosis *in vitro* as well as impeded tumor growth *in vivo*. MiR-204-5p directly bound to LINC00202 and HMGCR in RB cells, and LINC00202 functioned as a competing endogenous RNA in regulating HMGCR through competitively binding to miR-204-5p. More importantly, the regulation of malignant properties and glycolysis of RB cells mediated by LINC00202 could be reversed by abnormal miR-204-5p or HMGCR expression in RB cells. In all, LINC00202 promoted RB cell proliferation, glycolysis, and suppressed apoptosis by regulating the miR-204-5p/HMGCR axis, suggesting a novel therapeutic target for patients with RB.

## 1 Introduction

Retinoblastoma (RB) is an aggressive intraocular malignancy of infant and childhood, around accounting for 2.5–4% of all pediatric malignancies [1]. Early and effective treatment can preserve part of the visual function and achieve a longer survival time in children with RB; however, the optimal treatment time will be delayed if there is no timely diagnosis and treatment, thus resulting in a poor prognosis in children with RB [2,3]. Therefore, pinpointing the fundamental mechanism in the occurrence and development of RB and finding new targets to improve the treatment and prognosis of RB are necessary.

The molecular genetics and molecular-targeted therapies developing recently have provided promising strategies for improving the overall survival of children with RB [4]. As candidate therapeutic targets, long noncoding RNAs (lncRNAs) have been widely revealed to participate in various physical and pathological processes, such as cell differentiation, proliferation, apoptosis, and metabolism, thereby affecting the development and progression of cancers [5,6,7,8]. In RB, some lncRNAs have also been identified to play significant roles in the biological behaviors of cancers. For example, lncRNA-H19 promoted RB cell viability, metastasis while inhibited apoptosis through regulating runt-related transcription factor 2 (RUNX2) by binding to microRNA (miR)-143 [9]. LncRNA MALAT1 accelerated cell autophagy in RB by miR-124-mediated syntaxin 17 regulation [10]. LncRNA AFAP1-AS1 performed carcinogenic roles by facilitating RB cell tumorigenesis [11]. Up to date, it has been well-recognized that cancer cells suffer a metabolic reprogramming from oxidative phosphorylation to aerobic glycolysis that allows them to utilize glucose via anabolic fates for the enhancement of proliferation and other carcinogenesis-related features [12,13]. Thus, targeting cancer cell glycolytic metabolism may be a promising strategy for developing therapeutic interventions. In addition, abnormal lncRNAs expression has been documented to be related to the regulation of glycolysis [14,15]. LINC00202 is a novel identified lncRNA; Yan et al. demonstrated that LINC00202 was elevated in RB tissues and was related to the poor prognosis; besides, LINC00202 increased RB cell proliferation, migration, and invasion by binding to miR-3619-5p [16]. However, the functions of LINC00202 *in vivo* and glycolytic metabolism have not been clarified.

This study focused on the evaluation of LINC00202 function in RB carcinogenesis and aerobic glycolysis and explored the molecular mechanism underlying LINC00202 in the malignant properties and glycolysis in RB.

## 2 Materials and methods

### 2.1 Clinical specimens

Tumor specimens from 50 patients with RB and 50 normal retina samples from ruptured globes were obtained at Fenghua District People’s Hospital of Ningbo City and immediately preserved in liquid nitrogen. None of the subjects received chemotherapy or local radiotherapy before surgery. Besides that, the clinicopathological parameters of patients with RB, including age, gender, tumor size, affected eye, stages, and metastasis, were collected.


**Informed consent:** Informed consent has been obtained from all individuals included in this study.
**Ethical approval:** The research related to human use has been complied with all the relevant national regulations, institutional policies, and in accordance with the tenets of the Helsinki Declaration and has been approved by the research ethics committees of Fenghua District People’s Hospital of Ningbo City.

### 2.2 Cell culture and transfection

Human RB cell lines Y79 and HXO-RB44 were purchased from the Shanghai Academy of Life Science (Shanghai, China) and grown in the Roswell Park Memorial Institute (RPMI) 1640 medium (Gibco, Grand Island, NY, USA) supplemented with 10% fetal bovine serum (FBS; Gibco) and 1% antibiotic streptomycin/penicillin (Gibco). All cells were incubated at 37°C with 5% CO_2_.

To calculate the decrease of LINC00202, small interference RNAs (siRNAs) targeting LINC00202 (si-LINC00202), short hairpin RNA (shRNA) targeting LINC00202 (sh-LINC00202), and their negative control nonsense sequence (si-NC or sh-NC) were synthesized by GenePharma (Shanghai, China). For overexpression of LINC00202 and 3-hydroxy-3-methyl-glutaryl-coenzyme A reductase (HMGCR), the lentiviral LINC00202 expression vector (Lv-LINC00202) or lentiviral particles expressing pcDNA-HMGCR (HMGCR) and their negative control (Lv-NC or vector) were synthesized by Invitrogen (Carlsbad, CA, USA). The miR-204-5p mimics (miR-204-5p) and miR-NC, miR-204-5p inhibitors (anti-miR-204-5p) and anti-miR-NC were brought from RIBOBIO (Guangzhou, China). The transfection was carried out using Lipofectamine™ 2000 transfection reagent (Invitrogen).

### 2.3 Quantitative real-time polymerase chain reaction (qRT-PCR)

The isolation of total RNA was conducted using the TRIzol reagent (Invitrogen). Then, complementary DNA (cDNA) was generated using the PrimeScript reverse transcription reagent kit (Takara, Dalian, China). Next, quantitative PCR was performed on the 7500 Fast Real-Time PCR System using SYBR Green methods. Relative transcription expression was detected by the 2^−ΔΔCT^ method with glyceraldehyde 3-phosphate dehydrogenase (GADPH) and U6 small nuclear B noncoding RNA (U6) as the endogenous controls. The specific primer sequences were presented as follows: LINC00202: F 5′-TCAGTGGGTGTCCTCATTGGT-3′, R 5′-GCACAGTTTCATCCTCCTTCC-3′; miR-204-5p: F 5′-AACCUGAUCCCGUCUGAGAUUG-3′, R 5′-CCGGAUCAAGAUUAGUUCGGUU-3′; HMGCR: F 5′-TAGATTCGTTTCCCCAGG-3′, R 5′-TCGTTATCCAGAACCACC-3′; GADPH: F 5′-GATATTGTTGCCATCAATGAC-3′, R 5′-TTGATTTTGGAGGGATCTCG-3′; U6: F 5′-CTCGCTTCGGCAGCACA-3′, and R 5′-ACGCTTCACGAATTTGCGT-3′.

### 2.4 Cell proliferation analysis

Cell proliferation was conducted using cell counting kit-8 (CCK-8) assay. Transfected cells were seeded in 96-well plates overnight, then per well was added with 10 µL CCK-8 solution (Beyotime, Shanghai, China), followed by an incubation of 2 h. Finally, the absorbance at 490 nm was measured.

For colony formation assay, transfected cells (5,000/well) were seeded in 6-well plates with RPMI 1640 medium. After 21 days of incubation, cells were fixed with methanol and stained with 0.1% crystal violet, and the number of colonies was counted.

### 2.5 Cell apoptosis analysis

Transfected cells were collected and resuspended with the binding buffer, then the cells were interacted with 10 µL fluorescein isothiocyanate (FITC) annexin V and propidium iodide (PI) (BD Biosciences, San Jose, CA, USA). Finally, apoptotic cells were evaluated by Flow J software.

### 2.6 Measurement of extracellular acidification rate

The extracellular acidification rate (ECAR) was measured by using the Seahorse XF Glycolysis Stress Test kit. Transfected cells were grown in a Seahorse XF 96 cell culture microplate in the absence of glucose. After baseline measurements, saturating amounts of glucose, oligomycin, and the glucose analog 2-deoxyglucose (2-DG) were sequentially added into per well at indicated time points. Finally, the data were calculated using the Seahorse XF-96 Wave software.

### 2.7 Western blot

Total protein was extracted from tissues and cells using radioimmunoprecipitation assay (RIPA) buffer. Then, isolated protein was separated by 10% sodium dodecyl sulfate-polyacrylamide gel electrophoresis (SDS-PAGE) and electrophoretically transferred to polyvinylidene difluoride membrane (Millipore, Billerica, MA, USA). After that, immunoblotting was conducted using primary antibodies against Ki67 (1:1,000, ab16667, Abcam, Cambridge, MA, USA), pro-caspase-3 (pro-c3) (1:1,000, ab32150, Abcam), cleaved-caspase-3 (cleaved-c3) (1:1,000, ab2302, Abcam), lactate dehydrogenase A chain (LDHA) (1:3,000, ab135366, Abcam), HMGCR (1:5,000, ab174830, Abcam), and HRP-conjugated secondary antibody (1:1,000, ab9482, Abcam). GAPDH was as used as an endogenous control. Finally, protein signals were visualized using the chemiluminescence chromogenic substrate (Beyotime).

### 2.8 Dual-luciferase reporter assay

The LINC00202 and HMGCR 3′ UTR containing the wild-type (WT) or mutant (MUT) binding sites of miR-204-5p were ligated into the psiCHECK2 (Promega, Shanghai, China), respectively. Subsequently, Y79 and HXO-RB44 cells were cotransfected with these constructed reporter plasmids and miR-204-5p mimics or miR-NC using Lipofectamine™ 2000 (Invitrogen). Finally, the activities of luciferases were analyzed by using a dual-luciferase assay kit (Promega).

### 2.9 Xenograft experiments *in vivo*

Female BALB/c athymic nude mice (*N* = 6, 4–6 weeks old) were obtained from the National Laboratory Animal Center (Beijing, China). Y79 cells (1 × 10^7^) stably transfected with lentivirus containing sh-LINC00202 or sh-NC were subcutaneously injected into the flanks of the nude mice. Tumor size was measured and calculated every 4 days. On day 28, the mice were euthanized, and the tumor tissues were weighed and harvested for further study.


**Ethical approval:** The research related to animals’ use has been complied with all the relevant national regulations and institutional policies for the care and use of animals. This study was approved by the Animal Research Committee of Fenghua District People’s Hospital of Ningbo City and was conducted in line with the guidelines of the National Animal Care and Ethics Institution.

### 2.10 Statistical analysis

Data were presented as the mean ± standard deviation (SD). Significant differences were analyzed by one-way analysis of variance (ANOVA) or Student’s *t*-test using GraphPad Prism 7 software (GraphPad Inc., San Diego, CA, USA). The correlation analysis was performed using Spearman’s correlation test. All experiments were repeated three times. The *P*-value less than 0.05 indicated statistically significant.

## 3 Results

### 3.1 LINC00202 knockdown inhibits RB cell proliferation, glycolysis and induces apoptosis *in vitro*

**Figure 1:**
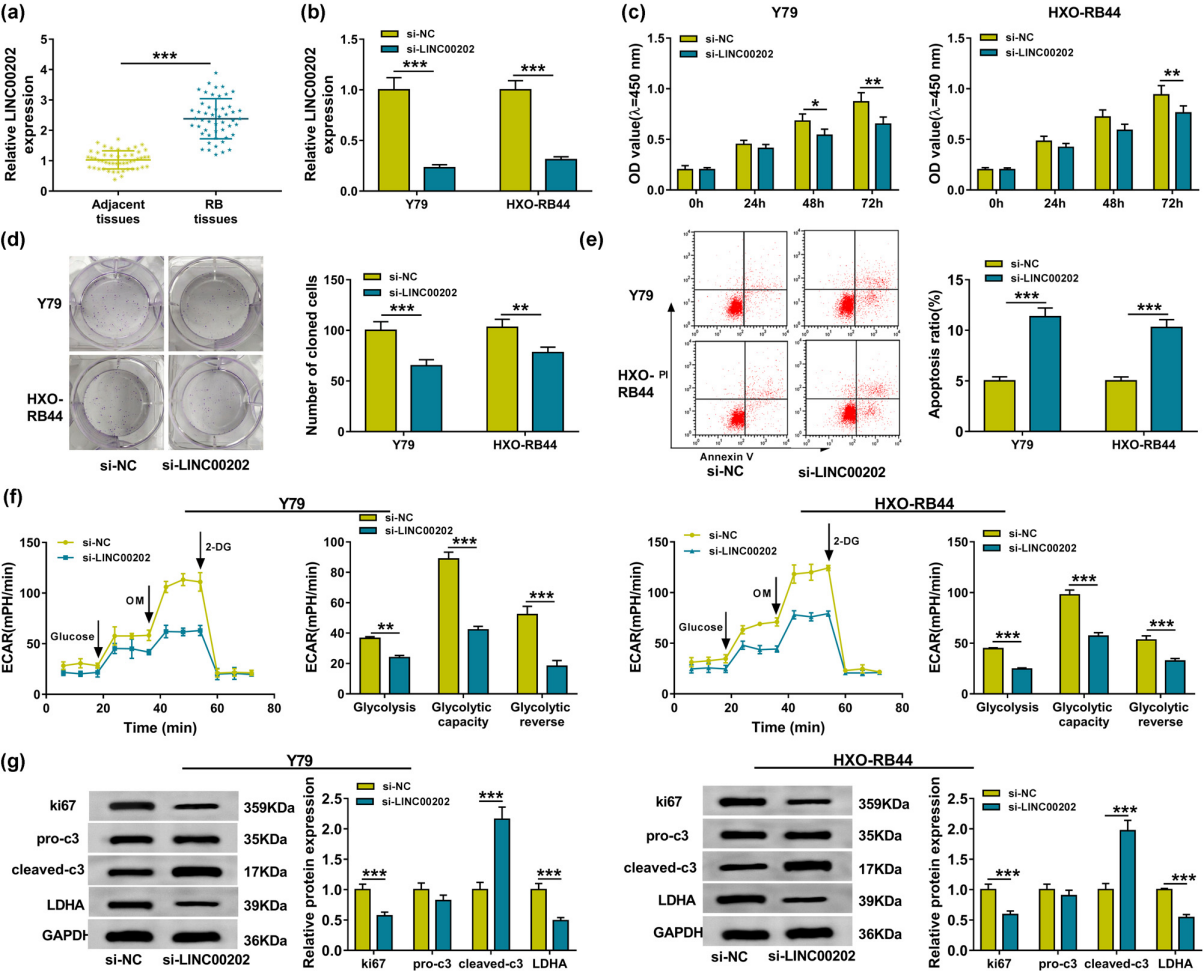
LINC00202 knockdown inhibits RB cell proliferation and glycolysis and induces apoptosis *in vitro.* (a) qRT-PCR analysis of LINC00202 expression in RB tissues and normal retina samples. Y79 and HXO-RB44 cells were transfected with si-LINC00202 or si-NC. After transfection, (b) qRT-PCR analysis of LINC00202 expression in RB cell lines, (c) cell proliferation analysis in Y79 and HXO-RB44 cells with CCK-8 assay; (d) colony formation analysis of Y79 and HXO-RB44 cell proliferation; (e) flow cytometry analysis of Y79 and HXO-RB44 cell apoptosis; (f) glycolysis stress test of ECAR in Y79 and HXO-RB44 cells; (g) western blot analysis of ki67, cleaved-caspase-3, pro-caspase-3, and LDHA expression in Y79 and HXO-RB44 cells. All experiments were repeated three times independently. *P < 0.05, **P < 0.01, ***P < 0.001.

The expression of LINC00202 was analyzed in RB tissues and normal retina samples, and qRT-PCR analysis showed that LINC00202 was elevated in RB tissues (Figure 1a), indicating that LINC00202 expression might be linked with the progression of RB. Additionally, patients with RB were grouped depending on the median level of LINC00202, and we found that higher LINC00202 expression was correlated with tumor size, stages, and metastasis in RB (*P* < 0.05, Table 1).

**Table 1:** Association of the relative expression levels of LINC00202 with clinicopathological parameters of patients with RB

Clinical parameter	Number of cases	Relative expression of LINC00202		
		Low	High	*P*-value
Total cases	50	24	26	
Age (years)				0.832
≤3	40	19	21	
>3	10	5	5	
Gender				0.963
Male	29	14	15	
Female	21	10	11	
Tumor size				0.004**
≤10 mm	23	6	17	
>10 mm	27	18	9	
Affected eye				0.273
Single eye	19	11	8	
Both eyes	31	13	18	
Stages				0.011*
Early stages (A–C)	28	9	19	
Advanced stages (D and E)	22	15	7	
Metastasis				0.026*
Yes	16	4	12	
No	34	20	14	

*P < 0.05.

**P < 0.01.

Next, the potential effects of LINC00202 on RB cell tumorigenesis were investigated; LINC00202 was knocked down in RB cell lines by using small interference RNAs; as expected, si-LINC00202 significantly reduced LINC00202 expression when compared with the si-NC group in Y79 and HXO-RB44 cells (Figure 1b). Subsequently, relative to the si-NC group, LINC00202 silence notably inhibited the proliferation of Y79 and HXO-RB44 cells (Figure 1c). Similarly, colony formation analysis also revealed that LINC00202 silence decreased the number of colonies formed in Y79 and HXO-RB44 cells (Figure 1d). Conversely, LINC00202 downregulation elevated the apoptosis of Y79 and HXO-RB44 cells (Figure 1e). Moreover, western blot analysis exhibited that the level of ki67 was lower, while pro-apoptotic proteins cleaved that caspase-3 level was higher in Y79 and HXO-RB44 cells transfected with si-LINC00202 than transfected with si-NC (Figure 1g). These data suggested that LINC00202 knockdown suppressed RB cell proliferation and induced apoptosis. In addition, we further detected the effect of LINC00202 on glucose metabolism in RB cells, and a glycolysis stress test was conducted. As shown in Figure 1f, with the treatment of glucose, oligomycin, or 2-DG, LINC00202 downregulation decreased the level of glycolysis, glycolytic capacity, and glycolytic reserve in Y79 and HXO-RB44 cells. Consistently, the LDHA level in Y79 and HXO-RB44 cells transfected with si-LINC00202 was inhibited when compared with the cells transfected with si-NC (Figure 1g). Thus, LINC00202 knockdown suppressed glycolysis in RB cells.

### 3.2 LINC00202 is a sponge of miR-204-5p in RB cells

**Figure 2:**
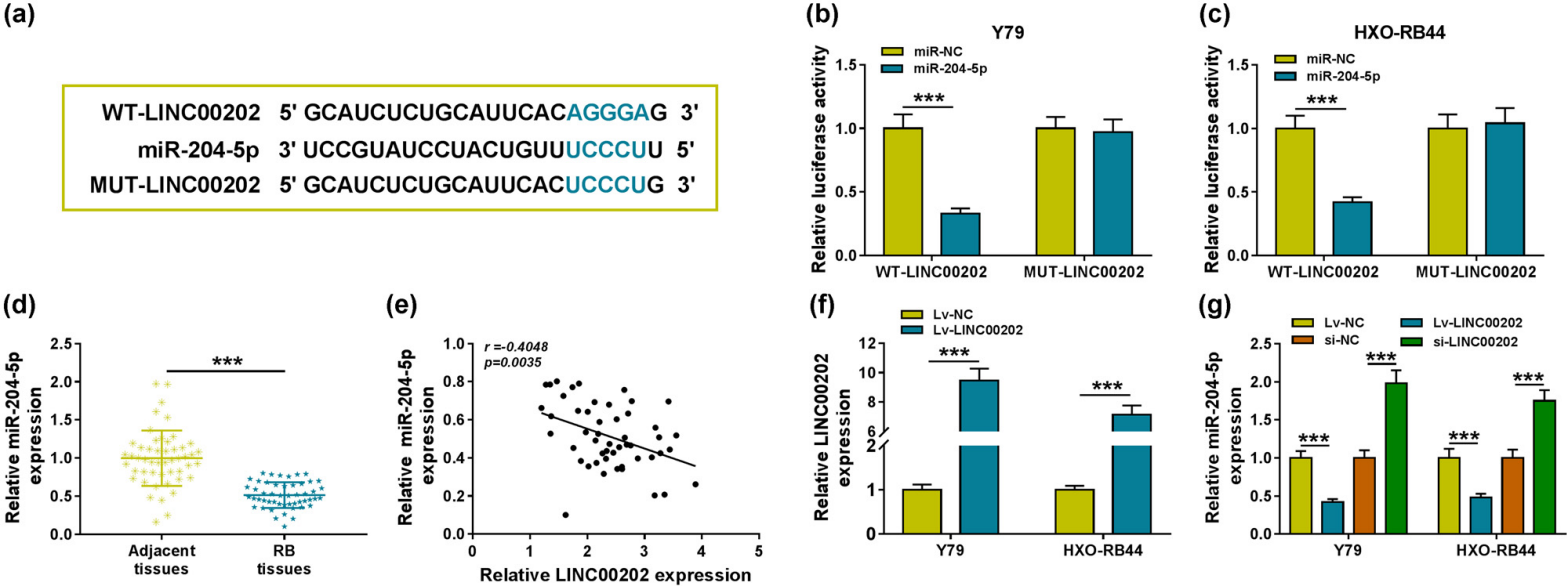
LINC00202 is a sponge of miR-204-5p in RB cells. (a) Schematic representation of the predicted binding sites between LINC00202 and miR-204-5p. (b and c) Dual-luciferase reporter assay in Y79 and HXO-RB44 cells cotransfected with WT-LINC00202 or MUT-LINC00202 and the indicated miRNAs. (d) qRT-PCR analysis of miR-204-5p level in RB tissues and normal retina samples (*N* = 3). (e) Spearman’s correlation analysis of miR-204-5p and LINC00202 expression in RB tissues. (f) qRT-PCR analysis of LINC00202 level in Y79 and HXO-RB44 cells transfected with Lv-LINC00202 or Lv-NC (*N* = 3). (g) qRT-PCR analysis of miR-204-5p in Y79 and HXO-RB44 cells transfected with Lv-LINC00202, Lv-NC, si-LINC00202, or si-NC (*N* = 3). *N* represents the test numbers, and the results represent the average of three independent replicates. *P < 0.05, **P < 0.01, ***P < 0.001.

The mechanism underlying LINC00202 in the malignant properties and glycolysis of RB cells was studied. Through searching the starBase3.0 program, we found miR-204-5p had the binding sites of LINC00202 (Figure 2a). Then, the reduction of luciferase activity in Y79 and HXO-RB44 cells cotransfected with WT-LINC00202 and miR-204-5p confirmed the direct interaction between LINC00202 and miR-204-5p in RB cells (Figure 2b and c). Subsequently, miR-204-5p expression in RB tissues and normal retina samples was investigated, and qRT-PCR analysis showed that miR-204-5p was decreased in RB tissues (Figure 2d), and a negative correlation between miR-204-5p and LINC00202 expression in RB tissues was investigated (Figure 2e). Furthermore, the expression of LINC00202 was upregulated in Y79 and HXO-RB44 cells by transfecting with Lv-LINC00202 (Figure 2f), and then, we found that the level of miR-204-5p was inhibited by LINC00202 overexpression but was elevated by LINC00202 downregulation in Y79 and HXO-RB44 cells (Figure 2g). Taken together, LINC00202 directly bound to miR-204-5p and negatively regulated its expression in RB cells.

### 3.3 LINC00202 promotes RB cell proliferation, glycolysis and inhibits apoptosis by interacting with miR-204-5p

**Figure 3:**
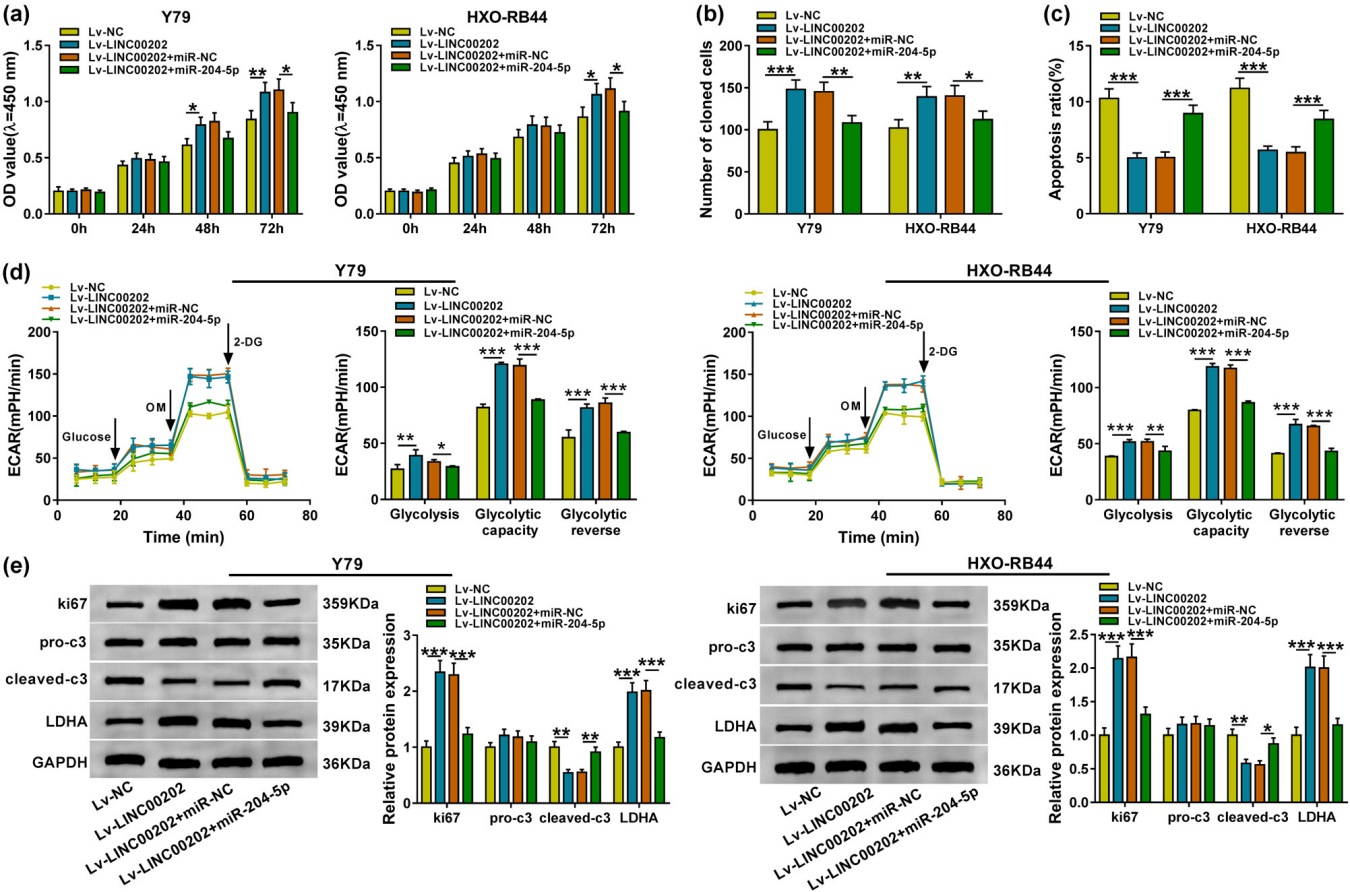
LINC00202 promotes RB cell proliferation and glycolysis and inhibits apoptosis by interacting with miR-204-5p. Y79 and HXO-RB44 cells were cotransfected with Lv-NC, Lv-LINC00202, Lv-LINC00202 + miR-NC, or Lv-LINC00202 + miR-204-5p. After transfection, (a and b) CCK-8 assay and colony formation analysis for the proliferation of Y79 and HXO-RB44 cells; (c) flow cytometry analysis for the apoptosis of Y79 and HXO-RB44 cells; (d) the glycolysis stress test of ECAR in Y79 and HXO-RB44 cells; (e) western blot analysis of ki67, cleaved-caspase-3, pro-caspase-3, and LDHA expression in Y79 and HXO-RB44 cells. The same experiment was repeated three times, and the average was taken. *P < 0.05, **P < 0.01, ***P < 0.001.

Based on the correlation between LINC00202 and miR-204-5p, we next explored whether LINC00202 functions dependent on miR-204-5p. Y79 and HXO-RB44 cells were cotransfected with Lv-NC, Lv-LINC00202, Lv-LINC00202 + miR-NC, or Lv-LINC00202 + miR-204-5p. Then, the results showed LINC00202 overexpression promoted cell proliferation (Figure 3a) and colonies formation (Figure 3b) but decreased apoptosis (Figure 3c) in Y79 and HXO-RB44 cells, while these effects could be attenuated by miR-204-5p upregulation (Figure 3a–c). Besides that, Lv-LINC00202-transfected Y79 and HXO-RB44 cells displayed significantly higher levels of glycolysis, glycolytic capacity, and glycolytic reserve when compared with the control, whereas these elevations were all abrogated by miR-204-5p mimic (Figure 3d). Also, the increase of ki67 level and LDHA as well as the decrease of leaved-caspase-3 level induced by LINC00202 overexpression in Y79 and HXO-RB44 cells were abolished by miR-204-5p upregulation (Figure 3e). Collectively, LINC00202 promoted cell malignant properties and glycolysis in RB via regulating miR-204-5p.

### 3.4 HMGCR is a target of miR-204-5p in RB cells

**Figure 4:**
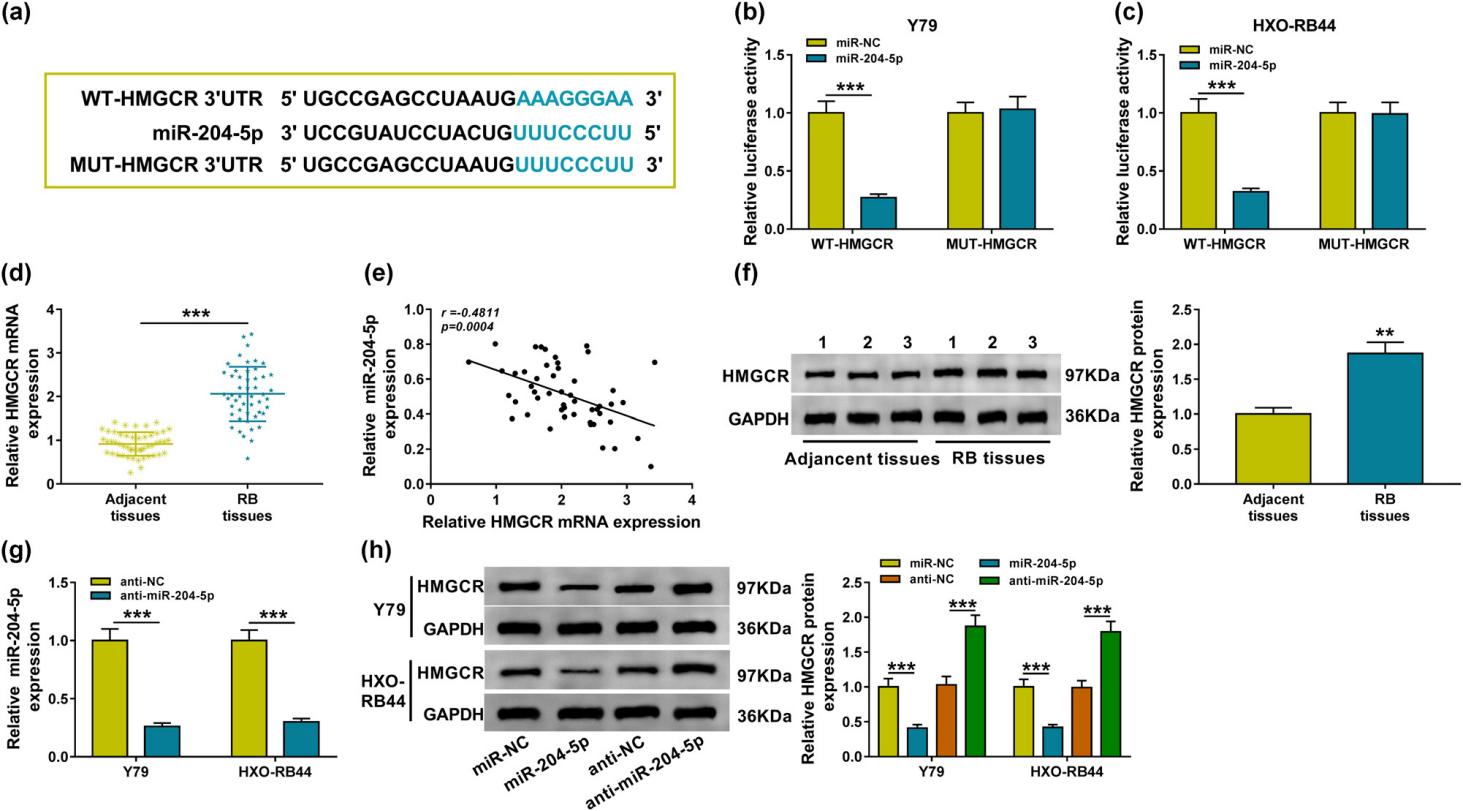
HMGCR is a target of miR-204-5p in RB cells. (a) Potential binding site of HMGCR in the miR-204-5p sequence. (b and c) Dual-luciferase reporter assay in Y79 and HXO-RB44 cells cotransfected with WT-HMGCR or MUT-HMGCR and the indicated miRNAs. (d) qRT-PCR analysis of HMGCR level in RB tissues and normal retina samples (*N* = 3). (e) Spearman’s correlation analysis of miR-204-5p and HMGCR expression in RB tissues. (f) Western blot analysis of HMGCR level in RB tissues and normal retina samples (*N* = 3). (g) miR-204-5p level in Y79 and HXO-RB44 cells transfected with anti-NC or anti-miR-204-5p using qRT-PCR analysis (*N* = 3). (h) Western blot analysis of HMGCR in Y79 and HXO-RB44 cells transfected with anti-NC, anti-miR-204-5p, miR-NC, or miR-204-5p (*N* = 3). *N* represents the test numbers, and the results represent the average of three independent replicates. *P < 0.05, **P < 0.01, ***P < 0.001.

By the prediction of the starBase3.0 program, HMGCR might be a potential target of miR-204-5p (Figure 4a). Then, a dual-luciferase reporter assay was performed, and a decline of luciferase activity in Y79 and HXO-RB44 cells cotransfected with WT-HMGCR and miR-204-5p was detected, indicating the direct interaction between HMGCR and miR-204-5p in RB cells (Figure 4b and c). In human tissues, HMGCR expression was elevated in RB tissues relative to the normal control tissues at mRNA and protein levels (Figure 4d and f) and was negatively correlated with miR-204-5p (Figure 4e). Additionally, after downregulating miR-204-5p expression in Y79 and HXO-RB44 cells using anti-miR-204-5p (Figure 4g), and we discovered that miR-204-5p upregulation decreased the level of HMGCR, while miR-204-5p downregulation increased the level of HMGCR in Y79 and HXO-RB44 cells (Figure 4h). These results suggested that miR-204-5p directly targeted HMGCR and negatively modulated its expression in RB cells.

### 3.5 LINC00202 functions as a competing endogenous RNA (ceRNA) in regulating HMGCR through competitively binding to miR-204-5p

**Figure 5:**
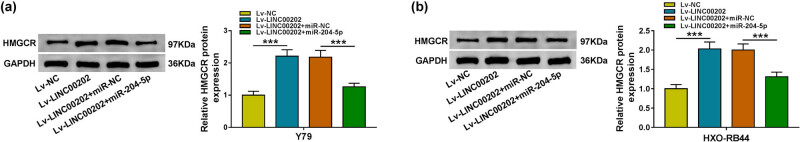
LINC00202 functions as a ceRNA in regulating HMGCR through competitively binding to miR-204-5p. (a and b) Western blot analysis of HMGCR expression in Y79 and HXO-RB44 cells transfected with Lv-NC, Lv-LINC00202, Lv-LINC00202 + miR-NC, or Lv-LINC00202 + miR-204-5p, experiments were performed three times, and the average was taken. *P < 0.05, **P < 0.01, ***P < 0.001.

Given that LINC00202 was a sponge of miR-204-5p and miR-204-5p targeted HMGCR in RB cells, we explored whether LINC00202 could regulate HMGCR via miR-204-5p. As shown in Figure 5a and b, we discovered that HMGCR expression was upregulated by LINC00202 overexpression, while this condition was reversed by miR-204-5p overexpression in Y79 and HXO-RB44 cells, indicating LINC00202 could regulate HMGCR through competitively binding to miR-204-5p.

### 3.6 LINC00202 silence represses RB cell proliferation, glycolysis and induces apoptosis by regulating HMGCR

**Figure 6:**
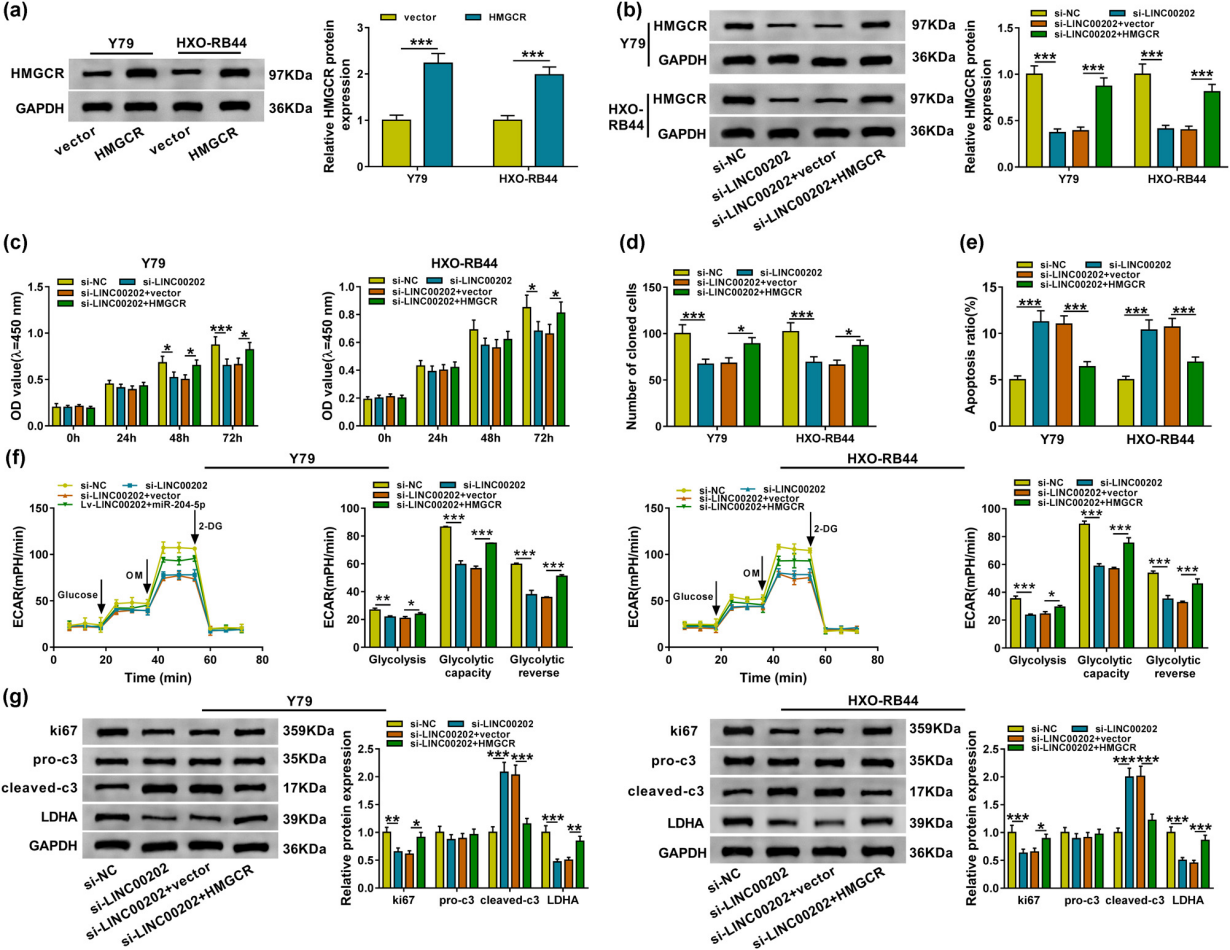
LINC00202 silence represses RB cell proliferation and glycolysis and induces apoptosis by regulating HMGCR. (a) Western blot analysis of HMGCR expression of HMGCR overexpression Y79 and HXO-RB44 cells. Y79 and HXO-RB44 cells were transfected with si-NC, si-LINC00202, si-LINC00202 + vector, or si-LINC00202 + HMGCR. After transfection, (b) Western blot analysis of HMGCR level in Y79 and HXO-RB44 cells; CCK-8 assay (c) and colony formation analysis (d) for Y79 and HXO-RB44 cell proliferation; (e) Y79 and HXO-RB44 cell apoptosis analysis by flow cytometry; (f) the glycolysis stress test of ECAR in Y79 and HXO-RB44 cells; (g) ki67, cleaved-caspase-3, pro-caspase-3, and LDHA expression analysis in Y79 and HXO-RB44 cells with western blot. Triplicate individual experiments were performed, and the average was taken. *P < 0.05, **P < 0.01, ***P < 0.001.

Considering LINC00202/miR-204-5p/HMGCR axis, we want to know whether HMGCR involved in LINC00202-mediated regulation on RB cell malignant properties and glycolysis. First, Y79 and HXO-RB44 cells were transfected with HMGCR, and HMGCR expression was notably increased in cells transfected with HMGCR when compared with that transfected with vector (Figure 6a). Subsequently, we found that HMGCR expression was decreased by LINC00202 deletion but was rescued by HMGCR transfection in Y79 and HXO-RB44 cells (Figure 6b), suggesting the potential involvement of HMGCR in the action of LINC00202 in RB cells. Next, functional experiment results showed that the effects of LINC00202 downregulation on Y79 and HXO-RB44 cell proliferation (Figure 6c and d), apoptosis (Figure 6e), and ki67 as well as the cleaved-caspase-3 level (Figure 6g) were all reversed by HMGCR overexpression. Besides that, HMGCR overexpression also abrogated LINC00202 knockdown-mediated inhibition on glycolysis, glycolytic capacity, glycolytic reserve (Figure 6f), as well as LDHA expression (Figure 6g) in Y79 and HXO-RB44 cells. Altogether, these results indicated that LINC00202 regulated cell malignant properties and glycolysis in RB by modulating HMGCR.

### 3.7 LINC00202 knockdown inhibits RB tumor growth *in vivo*

**Figure 7:**
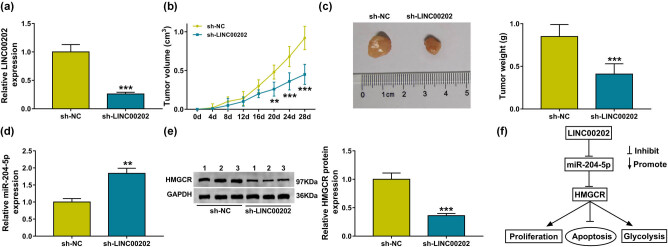
LINC00202 knockdown impeded tumor growth *in vivo.* (a) qRT-PCR analysis of LINC00202 level in Y79 cells transfected with sh-LINC00202 or sh-NC (*N* = 3). Tumor volume (b) and weight (c) analysis in xenografts from LINC00202 downregulation Y79 cells and negative control Y79 cells. (d) qRT-PCR analysis of miR-204-5p level in tumor mass from xenografts (*N* = 3). (e) Western blot analysis of HMGCR level in tumor mass from xenografts (*N* = 3). (f) Schematic diagram of LINC00202 regulating cell proliferation, apoptosis, and glycolysis in RB cells. *N* represents the test numbers, and the results represent the average of three independent replicates. *P < 0.05, **P < 0.01, ***P < 0.001.

To investigate the role of LINC00202 *in vivo*, Y79 cells were transfected with constructed lentiviral-sh-LINC00202 or lentiviral-sh-NC, and sh-LINC00202 transfection notably reduced LINC00202 expression compared with the sh-NC transfection (Figure 7a). Afterward, xenograft experiments exhibited that LINC00202 knockdown suppressed RB tumor growth *in vivo*, demonstrated by the decrease of tumor volume (Figure 7b) and weight (Figure 7c) in sh-LINC00202 group relative to the sh-NC group. Additionally, molecular analysis in tumor masses showed LINC00202 silence elevated miR-204-5p expression (Figure 7d) and reduced HMGCR expression (Figure 7e). Thus, we concluded that the LINC00202 knockdown might inhibit RB tumor growth *in vivo* by regulating HMGCR and miR-204-5p expression.

## 4 Discussion

Growing studies have reported the deregulation of lncRNAs in multiple tumors and the potential regulatory roles of lncRNAs in tumor cell survival, apoptosis, and proliferation [5,17]. LINC00202 is a newly identified lncRNA, and Liu et al. found that LINC00202 expression was linked with the overall survival (OS) time in patients with renal cancer [18]. Besides, the study of Yan’s team also exhibited that LINC00202 was implicated in the tumorigenesis of RB *in vitro* [16]. All these studies suggested the involvement of LINC00202 in cancer development. In this study, the function of LINC00202 in RB *in vitro* and *in vivo* was investigated. We found LINC00202 was elevated in RB tissues, LINC00202 deletion significantly inhibited cell proliferation and induced cell apoptosis in RB *in vitro*, while LINC00202 overexpression showed opposite effects. Besides that, LINC00202 downregulation reduced tumor volume and weight in xenograft mouse models. Thus, we concluded LINC00202 is an oncogene in RB.

Proliferative and cancer cells typically show a bias toward aerobic glycolysis rather than oxidative phosphorylation regardless of the oxygen availability to meet their energy demand for rapid growth [19]; relative to normal cells, this metabolic shift is conducive to the growth of cancer cells in tumor environments and also facilitates cancer malignancy by regulating oncogenic signaling [20,21]. In this study, the glycolysis stress test of ECAR was used to evaluate the effect of LINC00202 on RB cell aerobic glycolysis, and we found that LINC00202 deletion downregulated the level of glycolysis, glycolytic capacity, and glycolytic reserve in RB cells, and the expression of glycolytic enzyme LDHA was also reduced by LINC00202 silence. Based on our results, previous studies also uncovered the significant effects of lncRNAs on regulating glycolytic metabolism in cancers [22]. For example, lncRNA-p23154 facilitated cell invasion and metastasis of oral squamous cell carcinoma by the modulation of glucose transporter 1-mediated glycolysis [23]. LncRNA FGF13-AS1 suppressed cell glycolysis and stemness properties via insulin-like growth factor 2 mRNA binding proteins (IGF2BPs)/c-Myc pathway in breast cancer [24]. Taken together, our findings demonstrated that LINC00202 was an oncogene to promote RB progression by regulating cell proliferation, apoptosis, and aerobic glycolysis.

We further investigated the molecular mechanism of LINC00202 in RB. MiRNAs are classes of small noncoding RNAs that participate in the regulation of diverse biological process via targeting sequence of cellular and molecular pathways, and deregulations of these molecules are involved in various stages of RB [25,26]. MiR-204-5p is an important tumor suppressor and has been identified to involve in the development and progression of various cancers. For instance, miR-204-5p repressed tumor metastasis and immune cell reprogramming in breast cancer through modulating PI3K/Akt signaling [27]. MiR-204-5p contributed to cell apoptosis in prostate cancer via downregulating B-cell lymphoma-2 expression [28]. MiR-204-5p exerted antitumor activity by suppressing melanoma cell invasion [29]. More importantly, miR-204 was found to be decreased in RB, and miR-204 re-expression inhibited RB cell tumorigenesis and progression [2,30]. In this study, we confirmed miR-204-5p was a target of LINC00202, and miR-204-5p expression was negatively regulated by LINC00202 in RB cells. Additionally, the effects of LINC00202 upregulation on cell malignant properties and glycolysis could be reversed by miR-204-5p overexpression in RB. Thus, LINC00202 regulated RB progression by miR-204-5p.

HMGCR is a target of satin, widely available cholesterol-lowering drug, and is the rate-limiting enzyme of the mevalonate pathway for cholesterol synthesis [31,32]. Emerging studies have highlighted the oncogenic role of HMGCR in several cancer types, such as gastric cancer [33], breast cancer [34], and glioma [35]. In RB, it has been reported that HMGCR expression is increased in RB tissues and may be associated with the pathogenesis of RB; however, the biological functions of HMGCR in RB remain unknown [36,37]. In this study, HMGCR was found to be upregulated in RB tissues, and miR-204-5p targeted repressed HMGCR expression in RB cells. Besides that, HMGCR expression was positively correlated with LINC00202, and LINC00202 served as a ceRNA for miR-204-5p to modulate HMGCR expression. Subsequently, rescue assay showed HMGCR overexpression reversed LINC00202 silence-mediated repression on RB cell proliferation, glycolysis, and induction on apoptosis. Therefore, LINC00202 also regulated RB progression via HMGCR.

In conclusion, our findings demonstrated that LINC00202 functioned as an oncogene to regulate RB cell proliferation, apoptosis, and glycolysis by the axis of miR-204-5p/HMGCR (Figure 7f), which might be targeted for therapeutic benefits of RB.
